# The Influence of a Roux-en-Y Gastric Bypass on Plasma Concentrations of Antidepressants

**DOI:** 10.1007/s11695-023-06526-1

**Published:** 2023-03-22

**Authors:** Paul Garin, Lucie Favre, Nathalie Vionnet, Johanna Frantz, Chin B. Eap, Frederik Vandenberghe

**Affiliations:** 1 Unit of Pharmacogenetics and Clinical Psychopharmacology, Centre for Psychiatric Neuroscience, Department of Psychiatry, Lausanne University Hospital and University of Lausanne, Route de Cery 11b, CH-1008 Prilly, Switzerland; 2 Service of Endocrinology, Diabetes and Metabolism, Lausanne University Hospital, Lausanne, Switzerland; 3 Service of Liaison Psychiatry, Department of Psychiatry, Vaud University Hospital, Lausanne, Switzerland; 4 School of Pharmaceutical Sciences, University of Geneva, University of Lausanne, Geneva, Switzerland; 5 Center for Research and Innovation in Clinical Pharmaceutical Sciences, University of Lausanne, Lausanne, Switzerland; 6 Institute of Pharmaceutical Sciences of Western Switzerland, University of Geneva, University of Lausanne, Geneva, Switzerland

**Keywords:** Antidepressants, Roux-en-Y Gastric Bypass, Therapeutic drug monitoring

## Abstract

**Purpose:**

Roux-en-Y gastric bypass (RYGB) involves alterations of the gastrointestinal tract resulting in altered absorption. Patients with obesity have a higher prevalence of depression, and antidepressants are often prescribed. Alterations caused by RYGB could modify drug bioavailability and cause potential subtherapeutic plasma concentrations, increasing the risk of depressive relapse. The aim of this study was to describe the evolution of trough drug dose-normalized antidepressant plasma concentrations before and after RYGB.

**Materials and Methods:**

This naturalistic prospective case series considers patients with trough plasma concentrations in a 1-year timeframe before and after RYGB. Only antidepressants prescribed to at least three patients were included in the present study.

**Results:**

Thirteen patients (*n* = 12 females, median age 44 years, median BMI before intervention = 41.3 kg/m^2^) were included. Two patients were treated concurrently with fluoxetine and trazodone; the remaining patients were all treated with antidepressant monotherapy. Therapeutic drug monitoring (TDM) values for duloxetine (*n* = 3), escitalopram (*n* = 4), fluoxetine (*n* = 4), and trazodone (*n* = 4) before (median 4.7 weeks) and after (median 21.3 weeks) RYGB intervention were analyzed. Compared to preintervention, median [interquartile range] decreases in dose-normalized trough plasma concentrations for duloxetine (33% [− 47; − 23]), escitalopram (43% [− 51; − 31]), fluoxetine (9% [− 20; 0.2]), and trazodone (16% [− 29; 0.3]) were observed.

**Conclusion:**

This study shows a decrease in plasma antidepressant concentrations following RYGB. TDM before and after RYGB, in addition to close monitoring of psychiatric symptomatology, may help optimize antidepressant treatment after bariatric surgery. These results also highlight the need for prospective studies assessing the clinical evidence available through TDM in these patients.

**Graphical Abstract:**

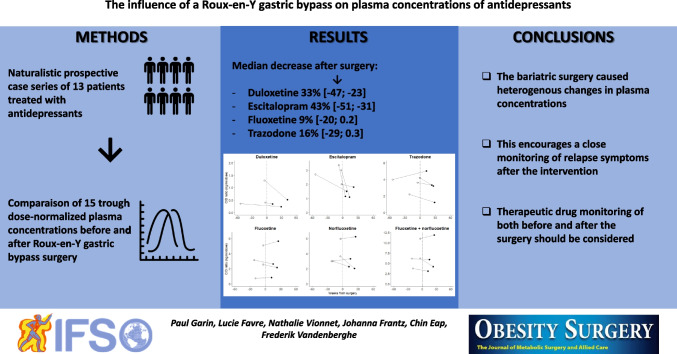

## Introduction

Obesity is on the rise worldwide, placing substantial pressure on health care systems. There is growing evidence that obesity and depression have a reciprocal association: obesity is more likely to increase the risk of onset of depression, while depression increases the odds of developing obesity [1]. Because of the higher prevalence of depressive disorders among patients with obesity, antidepressant prescriptions are common in this population. A recent systematic review concluded that depressive symptoms significantly decreased within the first 2 years following bariatric surgery. However, several longitudinal studies reported a symptomatology increase 2 years after intervention [2]. In addition to depressive symptoms, an increase in the suicide rate has also been observed in post-bariatric surgery patients [3]. Finally, a longitudinal database study of 400 patients showed that antidepressant discontinuation is less common than somatic medication discontinuation (60% decrease in statin use versus 19% for antidepressants) [4]. The reasons for increased depression symptoms in the long term remain unclear and could be multifactorial, including weight regain, insufficient weight loss, or decreased efficacy of antidepressants. Bariatric surgery may cause several mineral and vitamin deficiencies due to impaired absorption; however, little is known about drug absorption disorders.

The physiological mechanisms inducing impaired absorption are multifactorial and depend on the type of surgery. Roux-en-Y gastric bypass (RYGB) is one of the most commonly performed bariatric surgeries. The changes in intestinal and gastric architecture that it creates are likely to modify drug pharmacokinetics, particularly oral drug absorption. Altered gastric motility and emptying may negatively affect disintegration of oral solid formulation. In addition, reduced gastric volume may result in a lower volume of fluids to dissolve the drugs, and higher pH in the stomach could alter drug dissolution depending on their ionization state. The majority of oral drugs are absorbed through the small intestine, and thus, a decrease in absorption after RYGB may be expected [5]. Highly lipophilic drugs could undergo the same issue as fat-soluble vitamins, namely, reduced dissolution because of a delayed mix with bile salts and pancreatic secretions, causing a decrease in absorption and enterohepatic recirculation [6]. In addition to changes in the absorption phase, physiological changes associated with significant weight loss may also play a role in drug exposure. In patients with obesity, the increased amount of fat mass leads to an increased distribution volume of lipophilic agents such as psychotropic drugs.

Several reports have been published regarding the altered pharmacokinetics of psychotropic treatments such as haloperidol, lithium, lurasidone, long-acting paliperidone, or lisdexamfetamine [7–10]. Data regarding antidepressants are very scarce compared to the widespread use of this therapeutic class. Two cross-sectional studies examining sertraline and duloxetine pharmacokinetics in RYGB patients (sertraline *n* = 5, duloxetine *n* = 10) versus a matched non-surgical control group (sertraline *n* = 5, duloxetine *n* = 10) reported a significantly smaller area under the curve (AUC) and a shorter time to peak concentration (Tmax) for both drugs in the RYGB group [11, 12]. A prospective single-dose study found no significant AUC changes of 75 mg venlafaxine extended release and its active metabolite after RYGB in ten adults [13]. A case series in which four patients treated with escitalopram were followed before and at 2 and 6 weeks after RYGB showed a decline in plasma escitalopram concentrations after the intervention in all patients [14]. A case report also described a decrease of 50% in vortioxetine concentrations 91, 224, and 308 days after RYGB [15]. A case series on antidepressant concentrations during a 1-year follow-up post-RYGB in 12 patients showed a primary decrease in the first month followed by a normalized-to-increased AUC at 6 to 12 months post-surgery for selective serotonin reuptake inhibitors [16]. In addition to these published data, a recent naturalistic prospective study reported a significant decrease in post-bariatric surgery plasma concentrations of sertraline, mirtazapine, duloxetine, and citalopram (51%, 41%, 35%, and 19%, respectively) [17].

The aim of the present study was to describe the evolution of trough drug dose-normalized plasma concentrations before and after RYGB in patients treated with antidepressants.

## Methods

### Study Design and Participants

This is a case series of patients treated with antidepressants and undergoing RYGB (with a small gastric pouch (< 25 mL), an alimentary limb of 100 cm and a biliopancreatic limb of 50 cm).

According to international recommendations, a multidisciplinary team (e.g., a nutritional program and a psychologist) followed all patients before and after bariatric intervention [18, 19]. In addition, antidepressant and/or antipsychotic and/or anticonvulsant treatments were adapted based on plasma drug concentrations according to the national recommendation published by the Swiss Society for the Study of Morbid Obesity and Metabolic Disorders [20]. Approximately 1 to 3 months before intervention and as part of the psychiatric evaluation for bariatric surgery eligibility, the plasma drug concentration was measured in patients on psychotropic medication (antidepressants and/or antipsychotics). A second plasma drug concentration measurement was usually performed 3–6 months after intervention. These results were further transferred to the referral psychiatrist of the patient (out of the consultation obesity center) who assessed the usefulness of a treatment adaptation (treatment switch and/or dose adaptation). The exact time of the last drug intake and blood sampling, daily drug dose, treatment duration, comedications and plasma drug concentration recorded between 01.06.2017 and 31.12.2021 were extracted from patients’ electronic medical records. Patients who switched/stopped their antidepressant medication after bariatric intervention were excluded from the analysis. For patients with antidepressant associations, plasma drug concentrations were measured simultaneously for both antidepressants and were included separately in the analysis. To ensure that the drugs had reached steady state conditions, a minimum time interval of 5 days (30 days for fluoxetine) after changes in the dose was implemented. To avoid presenting individual data, only antidepressants prescribed to at least three patients were included in the present study.

### Plasma Drug Concentration Determinations

All blood samples were drawn in the morning under fasting conditions and were stored at -20 °C until routine analysis, which was conducted at a biweekly frequency. Plasma concentrations of trazodone, duloxetine, escitalopram, fluoxetine and its metabolite norfluoxetine were determined using liquid chromatography/mass spectrometry or ultra-performance tandem mass spectrometry methods. All methods were used on a routine basis in an accredited environment (ISO 15189).

### Statistical Analysis

Drug dose-normalized plasma concentrations (concentration/dose: C/D) were used since some patients required a dose change after the RYGB intervention. Due to the short half-life (mean *t*_1/2_ of 6.5 (standard deviation (SD) ± 1.6) h) and to be able to compare these results with published reference values, trazodone trough plasma concentrations were calculated at 12 h post-dose [21, 22]. Duloxetine trough plasma concentrations were calculated at 24 h post-dose using a mean *t*_1/2_ of 12 h (coefficient of variation 27%) [23]. The mean half-life of escitalopram of 10 and 30 mg/day varied between 29.0 h (SD ± 11.9) and 32.5 h (SD ± 14.2), respectively [24]. Given the usual dosages of 10–20 mg/day, the latter was calculated at 24 h post-dose, using a half-life of 30 h. The chronic administration of fluoxetine and norfluoxetine led to a half-life of 4–6 and 7–15 days, respectively [25]. Twenty-four hours post-dose plasma concentrations of fluoxetine and norfluoxetine were calculated using a half-life of 120 h (5 days) and 264 h (11 days), respectively. The comparison of C/D values before and after intervention was based on the sum of the parent compound and its active metabolite, as the therapeutic reference includes both compounds [26]. No statistical inferences were performed considering the small sample size; only medians and interquartile ranges (IQRs) are presented. All data analyses were performed with R (version 4.1.1) [27].

### Ethical Approval

All patients were previously included in the obesity cohort of Lausanne, an observational study investigating the long-term effects of bariatric surgery [28]. The Ethics Committee of CER-VD (N° 304/15) approved the study protocol, and written informed consent was obtained for all the included patients.

## Results

Thirteen patients were included; the large majority (92%) were female, with a median age of 44 years at the time of surgery. A median BMI of 41.3 kg/m^2^ was observed 4.7 weeks before intervention, when the first drug determination was completed (Table 1). This decreased to 34.7 kg/m^2^ 21.3 weeks after bariatric intervention (when the second drug determination was performed), corresponding to a median weight loss of 18.3%. The most frequent somatic comorbidities before intervention were non-alcoholic fatty liver disease (92.3%) and dyslipidemia (76.9%). All patients had a diagnosis of depression (ICD-10 F32.00 to F33.9) with, according to their medical records, four patients with anxiety and three with sleep disorder-associated symptoms. Three patients had an additional diagnosis of borderline personality disorder, obsessive–compulsive disorder and Tourette’s syndrome. Six patients (data not found for seven patients) had a depressive diagnosis established at least 7 years before intervention, and nine (data not available for four patients) had been treated with an antidepressant at least 1 year before intervention.

**Table 1:** Clinical and demographic parameters before and after bariatric intervention

	Pre-bariatric (*n* = 13)	Post-bariatric (*n* = 13)
Age et blood sampling (years) [IQR]	44.3 [40.5;53.1]	44.5 [41.0;53.9]
Body mass index (kg/m^2^) [IQR]	41.3 [38.0;50.0]	34.7 [31.9;40.3]
Weight change (%) [IQR]	− 18.3 [− 22.8; − 14.4]	
Gender:		
Female *n* (%)	12 (92.3%)	
Smoking status:		
Yes *n* (%)	2 (15.4%)	
Weeks between blood sampling and intervention [IQR]	4.7 [3.8;21.5]	21.3 [12.1;25.6]
Comorbidities at baseline: *n* (%)		
Arthritis	4 (30.8%)	
Chronic low back pain	2 (15.4%)	
Coronary artery disease	1 (7.7%)	
Dyslipidaemia	10 (76.9%)	
Gastro-oesophageal reflux disease	6 (46.2%)	
Hepatomegaly	6 (46.2%)	
Hypertension	5 (38.5%)	
Hyperuricemia	2 (15.4%)	
Non-alcoholic fatty liver disease	12 (92.3%)	
Type 2 diabetes	2 (15.4%)	

Median and interquartile ranges (IQR) are presented

**Fig. 1:**
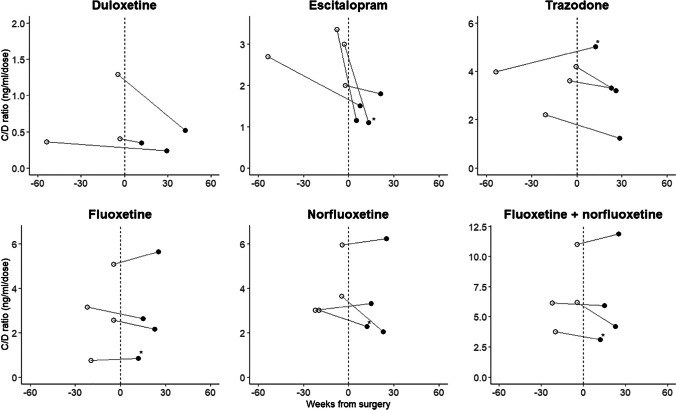
Standardized plasma concentrations/dose before and after RYGB intervention. * indicates patients with a dose modification

No comedications known to inhibit or induce CYP450 enzymes were identified either before or after RYGB. Of note, two patients were treated with trazodone associated with fluoxetine before and after bariatric intervention. Fluoxetine can increase plasma concentrations of trazodone through 3A4 enzymatic inhibition [29]. However, because this association was present before and after bariatric intervention, these observations were kept in the analysis. All included patients were treated with duloxetine (*n* = 3), escitalopram (*n* = 4), fluoxetine (*n* = 4), or trazodone (*n* = 4; one patient at a dosage for an antidepressant indication, 3 at a hypnotic dosage but associated before and/or after intervention with sertraline, escitalopram, or fluoxetine). All these treatments were taken once daily before and after intervention as tablets (escitalopram, fluoxetine, and trazodone) or capsules (duloxetine). The daily dosage of duloxetine remained identical before and after RYGB (Table 2). All patients on duloxetine were non-smokers. Blood samplings were performed between 19.5 and 24.5 h after the last drug intake; however, due to the short elimination half-life of duloxetine (12 h), all trough plasma concentrations were extrapolated at 24 h post-dose. A small decrease in the median standardized duloxetine plasma concentration in both pre- and post-bariatric observations (48.2 ng/mL and 30.9 ng/mL, respectively) resulted. For escitalopram, one sample was drawn 2.5 h after the last drug intake (close to the peak concentration, which is 2 h). A trough plasma concentration of 40 ng/mL was calculated based on an initial concentration of 67 ng/mL. The other samples were obtained under trough conditions. One escitalopram-treated patient received a dose increase from 5 to 10 mg/day after surgery. Median standardized escitalopram plasma concentrations decreased from 21.5 ng/mL (standardized C/D 2.1) to 16.1 ng/mL (standardized C/D 1.2) following the RYGB. All fluoxetine samples were obtained in trough conditions, and one patient received an increased dose from 40 to 60 mg/day after intervention. According to the long half-life of fluoxetine and norfluoxetine, minimal changes between plasma concentrations and standardized plasma concentrations were observed. Median standardized plasma concentrations (sum of fluoxetine and norfluoxetine) of 368 ng/mL (standardized C/D 6.1) and 303 ng/mL (standardized C/D 5.1) were observed before and after intervention. Only immediate release formulations of trazodone were prescribed once a day in the evening. One patient treated with 200 mg/day had his dose increased to 300 mg/day after intervention. The three remaining patients had a dose lower than or equal to 100 mg/day which was used to treat insomnia. These patients were also treated with fluoxetine (*n* = 2 also covered in the analysis) and sertraline-escitalopram (sertraline was switched to escitalopram after intervention, thus not eligible for analysis), indicating that insomnia is a symptom of depression. Blood samplings were performed between 10 and 19 h after the last trazodone intake; the latter were extrapolated at 12 h post-dose, resulting in a significant change in median plasma concentration. A baseline trazodone standardized plasma concentration of 291 ng/mL (standardized C/D 3.8) was observed, which decreased to 246 ng/mL (standardized C/D 3.3) following RYGB. As shown in Fig. 1, decreases in all escitalopram C/D ratios were observed after bariatric surgery, resulting in a median decrease in the C/D ratio of 43% [IQR − 51; − 31] (Table 2). Similarly, decreases in C/D ratios were observed for duloxetine (median decrease of 33% [IQR − 47; − 23]), which was, however, mostly driven by one observation. Three of the four patients treated with trazodone had a C/D decrease after intervention, resulting in a median decrease of 16% [IQR − 29; 0.3]. The total plasma concentration of fluoxetine + norfluoxetine remained comparable to prebariatric values (median decrease of 9%, IQR − 20; 0.2). All trough plasma concentrations remained in the reference range before and after RYGB, except for two patients treated with escitalopram who had post-surgery trough plasma concentrations of 13 and 11 ng/ml, respectively, which is close to the lower limit of the reference range (15–80 ng/ml) [26].

**Table 2:** Antidepressant treatment and therapeutic drug monitoring parameters before and after bariatric intervention

	Duloxetine (*n* = 3)		Escitalopram (*n* = 4)		Fluoxetine (*n* = 4)		Trazodone (*n* = 4)	
	Pre-bariatric	Post-bariatric	Pre-bariatric	Post-bariatric	Pre-bariatric	Post-bariatric	Pre-bariatric	Post-bariatric
Dose (mg/day)	120 [90.0;120]	120 [90.0;120]	10.0 [8.75;12.5]	10.0 [10.0;12.5]	40.0 [40.0;60.0]	60.0 [40.0;60.0]	100 [87.5;125]	100 [87.5;150]
Time between last drug intake and blood sampling (hours)	23.5 [22.0;23.5]	21.5 [20.5;23.0]	20.1 [12.4;24.7]	22.8 [20.8;24.8]	21.8 [20.5;25.0]	24.5 [24.0;24.5]	13.7 [13.4;15.2]	11.7 [10.6;13.2]
Plasma concentrations (ng/mL)	58.0 [51.0;69.0]	33.0 [31.5;43.5]	23.5 [18.8;37.0]	16.5 [14.0;19.2]	340 [245;382]	287 [218;350]	450 [396;572]	259 [152;597]
Standardized plasma concentrations (ng/mL)*^#^	48.2 [45.5;63.0]	30.9 [29.7;36.3]	21.5 [19.2;26.9]	16.1 [13.0;19.3]	368 [310;390]	303 [235;386]	291 [217;470]	246 [151;626]
Plasma concentrations/dose ratio^#^ (ng × day/mL × mg)	0.48 [0.42;0.91]	0.45 [0.36;0.48]	2.85 [2.53;3.09]	1.32 [1.14;1.57]	5.67 [4.21;7.22]	4.78 [3.64;6.78]	4.59 [4.50;4.70]	3.12 [2.39;3.79]
Standardized plasma concentrations/dose ratio (ng × day/mL × mg)^#^	0.40 [0.38;0.85]	0.35 [0.29;0.43]	2.14 [2.05;2.43]	1.24 [1.10;1.49]	6.14 [5.48;7.43]	5.06 [3.92;7.42]	3.80 [3.26;4.04]	3.26 [2.71;3.74]
Plasma concentrations/dose ratios change before and after intervention (%)^#^	− 33 [− 47; − 23]		− 43 [− 51; − 31]		− 9 [− 20;0.2]		− 16 [− 29;0.3]	

^*^ Plasma concentration standardized at 12 h post-dose for trazodone and at 24 h for escitalopram, duloxetine and fluoxetine

^#^Sum of fluoxetine and norfluoxetine

Median and interquartile ranges are presented

## Discussion

In the present study, the evolution of four antidepressant plasma concentrations in 13 patients before and after RYGB in a real-life setting were described. The prebariatric BMI in this sample is comparable to the obesity cohort of Lausanne undergoing RYGB, although the patients in the present study are slightly younger, suggesting that there is a negative impact of depression and/or other psychiatric comorbidities on BMI. All patients were treated with antidepressants that are known to have little effect on weight or even described as inducing weight loss and thus should not negatively affect weight loss after bariatric intervention [30, 31]. The present study indicates that RYGB has an important effect on the plasma concentrations of antidepressants. For escitalopram, the observed 43% decrease in C/D ratios post-RYGB is consistent with previous studies showing a decrease between 19 and 33% of post-RYGB surgery plasma concentrations [14, 17]. These data should warn of a possible decrease in the absorption of escitalopram, which is of clinical importance because of the widespread use of this antidepressant. A decrease in duloxetine plasma concentrations following RYGB is also in agreement with previous longitudinal and cross-sectional studies [12, 17]. The decrease in post-RYGB plasma concentrations can be partly explained by the presence of a slow-release capsule, although the effect of this formulation is a matter of debate, as shown with venlafaxine extended release [13]. Only small changes in fluoxetine were observed, which is comparable to a recent analysis reporting a non-significant increase in post-RYGB plasma concentrations [17]. The results of this study are the first data on the modification of trazodone absorption after RYGB. However, in the present study, trazodone was used mainly for a sedative purpose (immediate release and at low dosages), and the relevance of the present results for antidepressant use (usually at higher dosages and with extended-release tablets) should be cautiously evaluated.

Three patients (prescribed escitalopram, fluoxetine and trazodone) received a dose increase after the RYGB but before obtaining a post-RYGB plasma concentration. Thus, these dosage increases were based on clinical observations rather than therapeutic drug monitoring (TDM) data. The possibility of further dosage adaptations based on post-RYGB plasma concentrations cannot be excluded; however, the clinical impact of TDM on patient care is beyond the scope of this study. A twofold dose increase for escitalopram was not enough to obtain a similar-to-pre-RYGB plasma concentration, suggesting that a post-RYGB dose increase should be carefully evaluated and other strategies based on a modification of formulation should also be considered. Conversely, for the patients treated with trazodone and fluoxetine, an increase in post-RYGB plasma concentrations was observed, and no important C/D changes were observed. Although RYGB might alter the bioavailability of several oral antidepressants, the effect of bariatric surgery on depression and other psychiatric comorbidities should also be considered. The prevalence of depression decreases during the first 6 months, when an important degree of weight loss is observed, but rises again 2 to 3 years after intervention, when weight loss is plateauing [32]. In addition, inadequate weight loss and rebound weight gain are associated with an increase in depression and eating disorders [33, 34], highlighting the complex association between weight and psychiatric diseases. This suggests that TDM should be performed during the first months independently of the psychiatric evaluation, as symptomatology usually worsens during weight stabilization and two to 3 years after bariatric intervention. Through this method, and in case of a depressive relapse 2 to 3 years after intervention, the clinician can exclude a malabsorption phenomenon and directly adopt another therapeutic strategy (e.g., a compliance evaluation or a treatment switch).

Several limitations should be addressed. First, the small number of observations is a major limitation for reaching strong conclusions about the impact of RYGB on antidepressant absorption. This limitation did not allow us to statistically test the hypothesis of a change in trough drug dose-normalized plasma concentrations before and after RYGB. However, the comparison of patients with their pre- and post-intervention values should limit the interindividual variability and thus strengthen the validity of these observations. Second, only trough plasma concentrations are reported, which is not necessarily an indicator of decreased absorption, and thus, additional parameters such as AUC should be considered. Further studies should thus be conducted to confirm that trough plasma concentrations are a good indicator of impaired absorption under steady-state conditions after RYGB intervention. The real-world design also possibly introduces a selection bias, as several patients with an insufficient antidepressant response after bariatric intervention could have switched to another antidepressant before the post-bariatric dosage. However, this real-world setting may strengthen the generalization of these observations in a clinical setting. Finally, psychiatric evaluation was only performed to assess eligibility for bariatric intervention. Unfortunately, no systematic psychiatric evaluation (e.g., Beck depression inventory-II questionnaire) before and after intervention was available for this study. The usefulness of TDM as a predictor of depressive relapse in the post-bariatric period remains unknown and should be assessed in larger studies that also include an assessment of depressive symptomatology.

General recommendations regarding oral pharmacotherapy after bariatric surgery are often based on a modification of formulation, e.g., making them orally dispersible, liquid, or crushing pills if applicable, increasing/dividing the daily dose and switching to another psychotropic drug. Ideally, pharmacotherapy adaptation should be initiated in the preoperative period based on determination of basal psychotropic drug plasma concentration (if applicable) and assessment of the possibility of switching to liquid or sublingual melting forms. Shortly after surgery, clinicians should be aware of the appearance of withdrawal symptoms, and plasma concentrations should be obtained to exclude malabsorption [35]. Given that clinicians will increasingly be involved in the care of patients treated with psychotropic drugs and undergoing bariatric surgery, there is a need for well-powered prospective studies in a real-world setting to assess the evidence available through TDM in these abovementioned clinical recommendations.

## Conclusion

The influence of bariatric surgery on psychotropic drug absorption is an important issue, as the majority of patients do not discontinue their antidepressant treatment after surgery. This present case series of antidepressant plasma concentrations before and after RYGB suggests a lower absorption of the drugs 20 weeks after the operation. With few patients and varied results for each drug, these data highlight the importance of close monitoring of psychiatric symptoms before and after the operation and the need for more in-depth studies to improve clinical outcomes.

## Data Availability

The dataset analysed during the current study is not publicly available due the sensitivity of the human personal data involved, which requires specific precautions and limitations. The dataset is available on reasonable request.
